# Female Models in AI and the Fight Against COVID-19

**DOI:** 10.12688/f1000research.123599.1

**Published:** 2022-09-12

**Authors:** Claudia Guerrero, Santiago Mazuelas

**Affiliations:** 1 Basque Center for Applied Mathematics, Bilbao, 480009, Spain; 2 Ikerbasque, Basque Foundation for Science, Bilbo, 480009, Spain

**Keywords:** Women, AI, STEM, COVID-19, AI4FA COVID-19

## Abstract

Gender imbalance has persisted over time and is well documented in the fields of science, technology, engineering and mathematics (STEM) and singularly in artificial intelligence (AI). In this article we emphasize the importance of increasing the visibility and recognition of women researchers to attract and retain women in the AI field. We review the ratio of women in STEM and AI, its evolution through time, and the differences among disciplines. Then, we discuss the main sources of this gender imbalance highlighting the lack of female role models and the problems which may arise; such as the so called Marie Curie complex, suvivorship bias, and impostor syndrome. We also emphasize the importance of active participation of women researchers in conferences, providing statistics corresponding with the leading conferences. Finally, to support these views, we give examples of several prestigious female researchers in the field and we review their research work related to COVID-19 displayed in the workshop “Artificial Intelligence for the Fight Against COVID-19” (AI4FA COVID-19), which is an example of a more balanced participation between genders.

## Introduction

**Figure 1.**
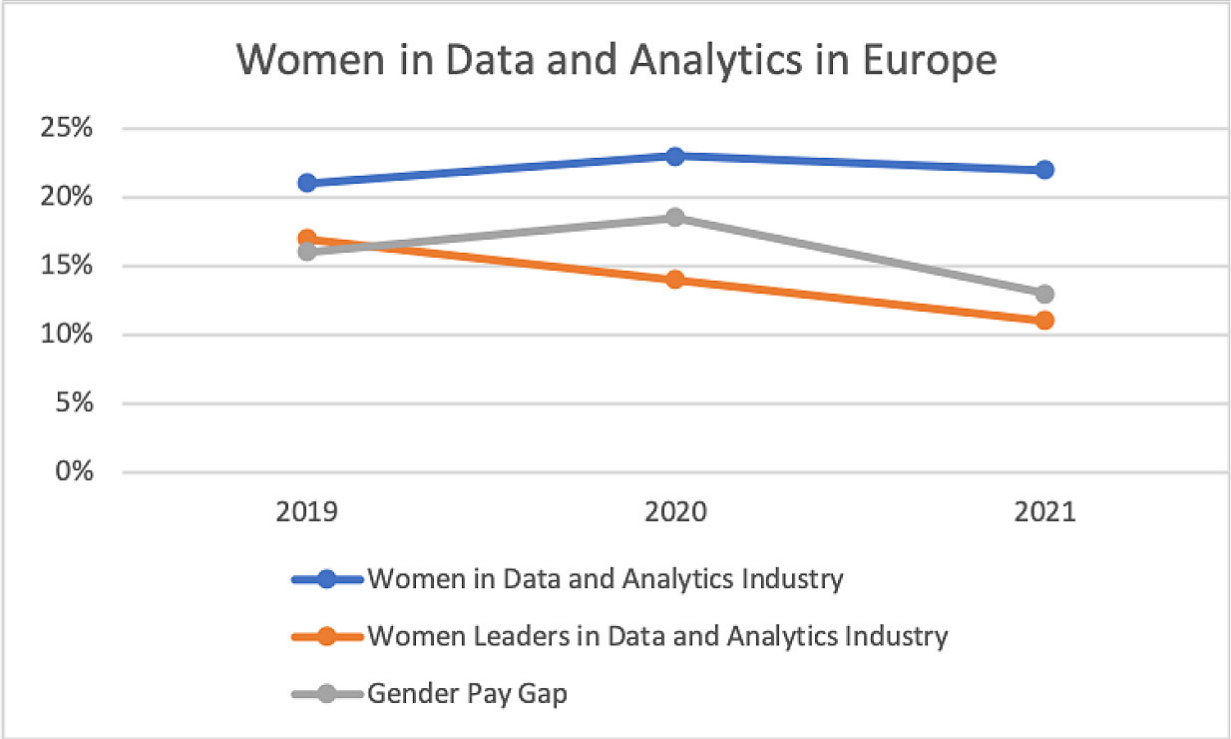
The ratio of women in data analytics industry in Europe. ^1^

Throughout history, women have played a prominent role in science, technology, engineering and mathematics (STEM) disciplines, however, their work has not always been duly recognized. The ratio of women in STEM positions has grown in the last decades, ^1^ but still they are underrepresented and growth rate has fallen in the last years (see Figure 1). Women constitute 47% of Europe workforce, ^2^ but the percentage of women scientists and engineers employed in high-tech sectors is only 22%. ^3^ The proportion of women also varies across STEM disciplines: in Europe the quota of women in tertiary education is 36% in physical sciences, 28% in mathematics and statistics, 21% in information and communication technology, and 27% in engineering. ^4^

In this opinion article, we review the ratio of women in artificial intelligence (AI), showing that the gender imbalance has decreased in the last years but progress is still too slow. We discuss the causes of gender imbalance in the field, placing special emphasis on the lack of female role models. We review related problems like the so-called Marie Curie complex, suvivorship bias, and impostor syndrome. Furthermore, we emphasize the leaky pipeline of women in STEM, highlighting the significant drop-out rates. In our opinion, to tackle gender imbalance in STEM we need to attract women to science and to retain researchers already in the field.

Next, we describe the participation of female researchers in conferences and the importance of events in which both men and women participate actively. We expose the workshop AI4FA COVID-19 as an example to support this view.

We highlight the career of four outstanding women researchers in AI: Prof. Concha Bielza, Prof. Emilie Chouzenoux, Prof. Mihaela van der Schaar, and Prof. Laure Wynants. Then, we review the contributions of those researchers for the fight against COVID-19 displayed in the workshop “Artificial Intelligence for the Fight Against COVID-19” (AI4FA COVID-19) carried out at BCAM in April 2022. Finally, we conclude that the gradual increase of women in AI is still too slow. Emphasis is placed on raising awareness in the community and making women in AI more visible and recognized as a measure to close the gender gap.

## Underrepresentation of women in AI

The gender gap remains in STEM disciplines, and is even more pronounced in AI and data analytics industry. According to Harnham diversity in data and analytics report, ^1^ in the last year the percentage of women in data industry has stayed the same or even dropped. The percentage of professional women in data analytics is 22% in Europe ( Figure 1) and 27% in the US and such inequality is accentuated in senior positions. Only 11% of leaders in the data industry in Europe are women and in Spain the ratio drops to 6%. The lack of female managers is reflected in a 13% pay gap with large fluctuations among countries. In the last year, France and Norway have reduced their pay gap to 5% and 7% while the Netherlands maintained a 24% gap and the UK doubled it from 7% to 13.5% in the last two years. ^1^ AI research also presents little gender equality. In 2019, only 13.8% of the scientific papers in AI were authored by women and the ratio of female co-authors has not increased in over 30 years. ^5^

**Figure 2.**
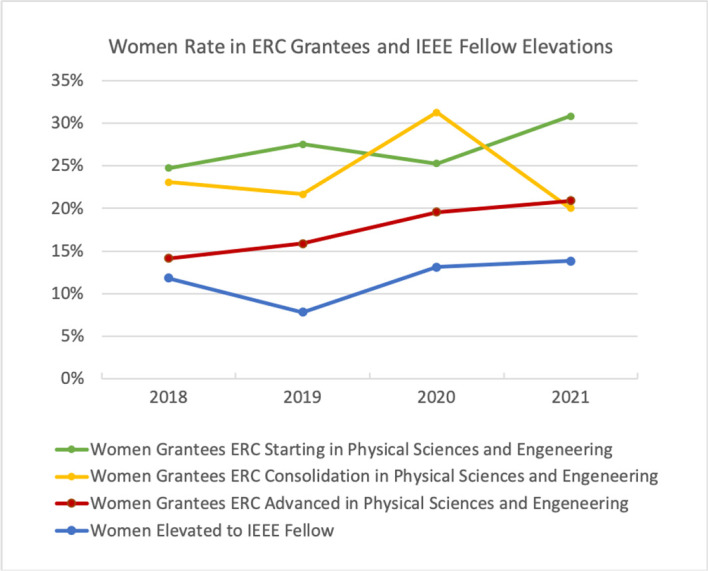
Women bestowed European Research Council Grants in Physical Sciences and Engeneering and elevated to IEEE Fellow. ^7^^–^^9^

Underrepresentation of women continues in grants and scientific societies. Only 23 women have won a Nobel Prize in physics, chemistry, and medicine since Marie Curie in 1903, compared to 606 men. ^6^ European Research Council (ERC) Grants bestowed to women have grown by 3% over the past four years, reaching 35% of female grantees by 2021. ^7^ However, the proportion of women is again significantly lower in the senior categories. For instance, in ERC Advanced Grants the rate of female grantees is only 25% ( Figure 2). ^7^ The percentage of women elevated to IEEE Fellow has slowly increased by 8% over the past two decades. In 2021 only 14% of IEEE Elevated Fellows were women ( Figure 2). ^8^^,^^9^

We are of the opinion that the reason for gender gap in STEM disciplines is not unique, but rather a set of deep social conceptions and biases difficult to synthesize. Studies show that two decisive factors are a masculinized view of science ^10^^,^^11^ and lack of a sense of belonging among women scientists to the intellectual community. ^11^ Both situations can be improved by making women in STEM more visible ^12^^–^^14^ and fostering collaboration both among women and among the intellectual community. ^11^^,^^12^

Lack of female visibility, in turn, is due not only to gender imbalance, but also to social biases. Female researchers denounce that their early works often only gain attention later in their careers, in stark contrast to their male peers. ^5^ We consider that a system that only pays attention to female researchers when they are superlative talented is doomed to discourage present and future female researchers by fostering the Marie Curie complex. This term refers to the impact of highlighting exclusively exceptionally brilliant women scientists, such as the first women in earning a Nobel Prize, Marie Curie. It leads to believe that successful female STEM careers are exceptional and unattainable. Lack of attainable role-models might promote unrealistic high standards which may lead to frustration, lack of a sense of belonging, impostor syndrome and, ultimately, not to pursue a scientific career or give up on it.

Bringing more diversity and equality into STEM is crucial for three main reasons. First, the participation of women is always positive to bring different perspectives into the field, and can contribute to increase innovation. Second, according to the European Institute for Gender Equality, closing gender gap in STEM would increase overall productivity and labor market activity fostering economic growth. ^15^ Last, it is already a fact that AI and technological advances are redefining our future as a society. If women do not participate in that transition, womens’ perspectives in our collective future would be lost.

To achieve a more gender-balanced scenario in STEM, it is clear that a primary goal is to attract women to STEM disciplines, but the work does not stop there. To bring more gender equality into STEM, we need women to stay in science. The second goal is to avoid the leaky pipeline of women leaving STEM disciplines. The leak of women in STEM is confirmed by the gap between women studying STEM tertiary education in contrast to the percentage of women in the STEM workforce or in research. Furthermore, numerous studies find that women leave STEM significantly more than men. ^16^^–^^19^ For example, in the US 24% of women leave full-time STEM employment in contrast to 16% of men, i.e., 50% more. When we focus on researchers who had become parents the figures increase to 23% for men and a devastating 43% for women. ^20^

We consider it critical to highlight the leaking of women in the STEM pipeline. This implies that while improving the visibility of women in the field is necessary, it is not sufficient. Focusing only on successful women who have been able to stay in science might spawn survivorship bias. This term refers to the logical error of concentrating on those that past some selection process and overlooking those that did not, which may lead to false conclusions. We want to emphasize the need to address gender gap in STEM as an structural problem that needs to be tackled from the root. We believe that the first step is to make the whole scientific community aware of the problem to be able to work on it collectively.

## Female models in AI

Despite gender imbalance, there are many women pioneering AI research around the globe. There are organizations to support them and foster collaboration among women in the field. For example, the initiative Women in AI (WAI) seeks to bring awareness and knowledge through education, like the educational program for training and mentoring Wai2GO, events, like Women in AI Awards, and blogging. https://www.widsconference.org inspires and educates data scientists worldwide and supports women in the field by means of the annual https://www.widsconference.org/conference.html, annual datathons dedicated to different topics of social interest, podcasts, and an education outreach program. Women in Machine Learning (WiML) enhances the experience of women in machine learning by organizing an annual workshop co-located with NeurIPS, small events, a mentoring program for Ph.D. students and a directory and profiles of women in machine learning. ^21^

We find these initiatives of great importance to support women in AI and improve their visibility. Nevertheless, we find a fundamental flaw that is repeated all over these kind of events and initiatives: the public is almost exclusively female. We think that fostering collaboration among women is vital, but we should be careful not to call for gender apartheid in the way. We believe that, ultimately, the only effective way to achieve gender balance is to approach the problem collectively. We cannot change how the STEM disciplines operate without more than half of the STEM community. For this reason we believe that promoting collaboration both among women and among each and every person in STEM regardless of gender is critical.

In our opinion, scientific conferences and workshops should include people of all genders as audience and speakers. It is this second part that, unfortunately, is most often neglected in non-exclusively female initiatives. A study conducted in 2018 showed that only 12% of researchers published in the leading AI conferences (Neural Information Processing Systems, International Conference on Machine Learning, and International Conference on Learning Representations) are women. ^22^ This number contrasts with the 22% of women working in AI.

Next, we describe the workshop AI4FA COVID-19 as an example of a more gender-balance collaboration in AI events. This event counted with a 33% of female speakers and a 100% of female keynote speakers. The workshop received the participation of four women leading AI research for the fight against COVID-19 as keynote speakers: Prof. Concha Bielza, Prof. Emilie Chouzenoux, Prof. Mihaela van der Schaar, and Prof. Laure Wynants. These researchers, besides working on cutting-edge research on different collaborative projects, are pioneers in their fields. In addition, they are a cross-section of women’s leadership in AI, at different points in their careers and with varying degrees of experience and background. In the workshop they shared their work with a diverse audience of researchers. In what follows, we introduce them and the work they presented on this workshop.

Professor Bielza is Full Professor of Statistics and Operations Research in the AI Department at the Polytechnic University of Madrid. She has received multiple awards for her work, highlighting the Research Award for Significant Contribution in the Field of Machine Learning granted in 2020 by Amity University. In 2021 she was appointed Member of the Scientific Advisory Board of NorwAI (Norwegian Research Center for AI Innovation) and she is a Member of the Transfer Committee of the Spanish Royal Society of Mathematics and a Member of the Academic Council of ValgrAI (Valencian Graduate School and Research Network of Artificial Intelligence). Along her career, Concha has pointed out the importance of female participation in AI to define our own future. In addition, she emphasizes the importance of the participation of men in the joint responsibility in contributing to close the gender gap. ^23^

Professor Chouzenoux is a Researcher at Inria Saclay within the project team OPIS (Optimization for large scale biomedical data), in the Center for Digital Vision of University Paris Saclay. She is the principal investigator of the ERC Starting Grant MAJORIS initiated in 2020. She is also an Associated Editor for the IEEE Transactions in Signal Processing, and SIAM Journal on Mathematics of Data Science. Regarding gender gap in STEM, Emilie spots that a major factor is lack of information and the impostor syndrome. She proposes introducing girls from an early age to the careers of successful researchers in order to broaden the options for the future they envision. She also emphasizes that the fight for women’s participation in science could benefit from being considered more widely. Therefore, it is important to educate young girls and boys in a joint dissemination of scientific culture, at school and family sphere. ^24^

Professor van der Schaar is the John Humphrey Plummer Professor of Machine Learning, Artificial Intelligence and Medicine at the University of Cambridge and a Fellow at The Alan Turing Institute in London. In addition to leading van der Schaar Lab, she is the founder and director of the Cambridge Centre for AI in Medicine. She has received numerous international awards and recognition for her work, highlighting the Oon Prize on Preventative Medicine from the University of Cambridge, three IBM Faculty Awards, the IBM Exploratory Stream Analytics Innovation Award, the Philips Make a Difference Award, Star in Computer Networking and Communications by N2Women, Royal Society Wolfson Research Merit Award, and the IEEE Darlington Award. She was elected IEEE Fellow in 2009 and she is the most-cited female AI researcher in the U.K. ^5^ During her career, Mihaela has organised several outreach activities dedicated to empowering women in engineering and computer science. To address gender imbalance among AI workers and researchers, Prof. van der Schaar proposes to show the creative and human aspect of AI and ML to the public, especially to women. ^25^

Professor Laure Wynants is an Assistant Professor of Epidemiology at Maastricht University and KU Leuven. She is an Associate Editor for BMC Diagnostic and Prognostic Research, member of the International Society for Clinical Biostatistics, and member of STRATOS’ (STRengthening Analytical Thinking for Observational Studies) topic group on the evaluation of diagnostic tests and prediction models. She wrote one of the most read papers in Statistics in Medicine in 2019. ^26^ In addition, she has received multiple awards including the Edmond Hustinx Prize for Science. Laure has pointed out often the need to increase the gender-balanced in science, for instance, she declared that, after more than 10 years in the field, she was glad to attend for the first time a completely female panel at the Winter School of the University of Padova. ^27^

## Workshop AI4FA COVID-19

As mentioned above, the workshop AI4FA COVID-19 is an example of a more gender-balance in AI events with a 33% of female speakers and a 100% of female keynote speakers.

There are indeed women doing cutting-edge AI research and it is vital to recognize their work and give them much more visibility at all career levels to bring more women into the field. The workshop AI4FA COVID-19 counted with the participation of four excellent female keynote speakers doing remarkable research. ^28^^,^^29^ It was hosted by the Basque Center for Applied Mathematics (BCAM) in collaboration with AXA Research Fund and the Basque Government. The workshop was held from the 6 *^th^* to the 8 *^th^* of April 2022 and it was part of the project “Early prognosis of COVID-19 infections via machine learning” funded by AXA Research Fund under the Exceptional Flash Call “Mitigating risk in the wake of the COVID-19 pandemic”, and the project “Mathematical Modelling Applied to Health” funded by the Basque Government.

**Figure 3.**
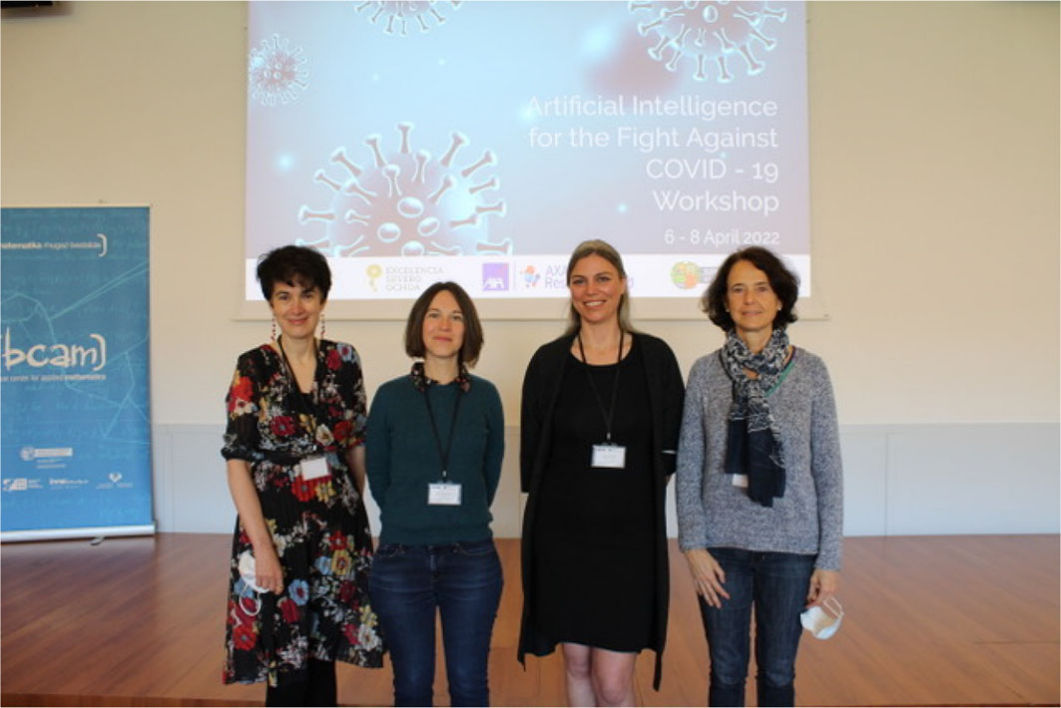
Keynote speakers at AI4FA COVID-19 workshop. ^30^ From left to right: Prof. Mihaela van der Schaar, Prof. Emilie Chouzenoux, Prof. Laure Wynants and Prof. Concha Bielza.

The workshop featured presentations by keynote speakers: Prof. Concha Bielza, Prof. Laure Wynants, Prof. Mihaela van der Schaar, and Prof. Emilie Chouzenoux ( Figure 3). The four keynote speakers presented leading-edge investigation of AI techniques applied to the fight against COVID-19 pandemic. They are women pioneering in this area of research.

Prof. Bielza opened the workshop with the session “Interpretable machine learning applied to COVID-19.” She presented her work and preliminary analyses with data from patients in hospitals of the six waves of the pandemic in Madrid. Her investigation puts a special spotlight on model interpretability and human-in-the-loop solutions by means of Bayesian networks ^31^ and decision trees. ^32^

Prof. Wynants presented the talk “A journey through the disorderly world of diagnostic and prognostic models for COVID-19: a living systematic review.” She leads the international consortium conducting a living systematic review of diagnostic and prognostic models for COVID-19. ^33^ They have screened over 126000 records, 412 studies and 731 prediction models. It already has over 2000 citations, and has been picked up by policymakers, including the European Commission and the World Health Organization. She detected issues with applicability and methodological biases in the majority of models, and identified a small number of usable models.

Prof. van der Schaar presented her work “Covid and AI: Unexpected challenges and lessons.” She emphasized what she and her team learned from working with clinicians. She noted that explainability means something different for them than for AI professionals and that explainable AI is vital in clinical applications. Mihaela addressed the explainability problem by using symbolic metamodeling. ^34^^,^^35^ It consists on finding a whitebox model, defined by an explicit function, for an input blackbox model.

Prof. Chouzenoux participated in the workshop with the session “Data Science and Artificial Intelligence for healthcare: COVID-19 use case.” She presented an AI model developed within the collaborative project ScanCovIA. The model gives accurate COVID-19 prognosis decision to help clinicians. It processes computerized tomography imaging features together with selected clinical and biological biomarkers. ^36^^,^^37^

The main conclusion of the workshop was that quality AI models for fight against COVID-19 need quality data. Therefore, AI work starts in health care services. It is key to work with clinicians, learn about their needs and explain them the data that will make it possible to meet those needs.

Projects like AI4FA COVID-19 workshop help in celebrating leading women researchers in AI, and in put a face on them. In this way, both the representation of women in the field and the disparity in the recognition they receive can be improved.

## Conclusions

The number of women working and researching in AI is increasing, however, there is still much work to be done to achieve parity in representation and recognition. There is a clear need to take action to attract and retain women in artificial intelligence (AI) in order to close the actual gender gap. We believe that it is necessary to make women in the field more visible to address the problem. It is essential to give visibility to women researchers in AI, with special emphasis on the early career, as this is the period in which there has been the most inequality in public recognition. Providing spaces for female AI figures to speak up and disseminate their research and careers can be an important step in closing the gender gap.

Furthermore, we believe that the first step should be to raise awareness of the problem and its various manifestations among the entire scientific community working in AI. We propose to focus on the following issues: less recognition for female research, specially in early careers, higher female drop-out rates, Marie Curie complex, suvivorship bias, and impostor syndrome.

We look at the percentage of female researchers published in leading conferences as a measure of gender-balance in the field, finding that only 12% of authors are women. We insist on the importance of events that bring together men and women in AI to share their research with gender parity both among the audience and the speakers, and we present AI4FA COVID-19 workshop as an example of such an event.

The outstanding women researchers; Prof. Bielza, Prof. Chouzenoux, Prof. van der Schaar, and Prof. Wynants, provide a remarkable example of cutting-edge AI research lead by women. The AI4FA COVID-19 workshop was honored to have the participation of these four keynote speakers to present their work on AI for the fight against COVID-19. We highlight the workshop as an example of the kind of activities that can help attract and retain more female researchers to the field.

We maintain that raising awareness in the STEM community and improving visibility and recognition of female researchers at every career level are key aspects to attract more women into STEM and break the current cycle of women leaving the field.

## Data availability

No data are associated with this article.
