# Supplementary material for: Female Models in AI and the Fight Against COVID-19
Source: F1000Res. 2022 Sep 12;11:1037. [Version 1] doi: 10.12688/f1000research.123599.1 (PMC11409909; doi:10.12688/f1000research.123599.1)
Supplement: Peer review history

# Reviewer report: Reviewer response for version 1

**DOI:** 10.5256/f1000research.135724.r187751

**Published:** 2024-09-16

**Authors:** Eva Cernadas

**Affiliations:** 1 University of Santiago de Compostela, Santiago de Compostela, Galicia, Spain

This paper analyses the gender imbalance in the artificial intelligence (AI) field. It summarizes the ratio of data analytics industry in the last years, the evolution of AI women researches in international conferences, as a leaders, as authors of scientific papers, wining the Nobel Prize, in the European Research Council (ERC), etc. From this analysis, the authors try to explore the causes of the current gender gap in STEM disciplines and, specifically, in the AI field.

 They conclude that the gender gap in STEM is a structural problem that needs to tackled from the root. They propose some initiative to make the whole scientific community aware of the problem in order to solve it.

 One of this initiative is the organization of the workshop AI4FA COVID-19. This event counted with 33% of female speakers and 100% of female keynotes speakers (Dr. Concha Bielza, Emilie Chouzenoux, Mihaela van der Schaar and Laure Wynants), which are pioneers researchers in AI. With events like this, we can improve the visibility of women in the scientific community and in the society.

 Although the authors describe an initiative to improve gender imbalance in AI, I think they can mention some strategies to attract female students to the high education in the STEM disciplines and how to promote female STEM vocation in the non-university students.

Is the topic of the opinion article discussed accurately in the context of the current literature?

Yes

Are arguments sufficiently supported by evidence from the published literature?

Yes

Are all factual statements correct and adequately supported by citations?

Yes

Are the conclusions drawn balanced and justified on the basis of the presented arguments?

Partly

Reviewer Expertise:

computer vision, machine learning, artificial intelligence, gender

I confirm that I have read this submission and believe that I have an appropriate level of expertise to confirm that it is of an acceptable scientific standard.

# Reviewer report: Reviewer response for version 1

**DOI:** 10.5256/f1000research.135724.r171781

**Published:** 2023-11-30

**Authors:** Briga Hynes

**Affiliations:** 1 University of Limerick, Limerick, County Limerick, Ireland

The aim of this article is to review the ratio of women in STEM and AI, its evolution through time, and the differences among disciplines and gender and an assessment of the sources of this gender imbalance. It makes suggestions on the importance of active participation of women researchers in conferences, providing statistics corresponding with the leading conferences *.*

 Overall, this is an interesting and timely opinion article given the heightened increase in both AI and the continued calls in policy and practice to increase the participation of females across a number of STEM related activities both in employment, in commercialising research and in the startup and entrepreneurship domains.

 The following review provides feedback on the strengths of the paper and provides suggestions on areas of development in the editing of the piece.  It is a challenge for authors to manage word count when they are so familiar with and passionate about sharing their learnings and suggestions for advancement on a topic and thus, I am sure the answers to the comments below are readily available to the authors.

 The introduction section would benefit from including descriptors or the parameters for the key themes being investigated to ensure clarity and signalling to the reader as to the specifics of what are broad topics.  For instance:

- AI - as it is an ever-changing concrete and a term used interchangeably and multidimensional – so is it AI in general or AI as part of the researcher in general population as is AI more specifically relevant to certain cohorts.
- Researcher –the researcher population is broad in nature - Is this paper examining researchers working in or within employment only or is the data relating to start up or the commercialisation of research.
- Is the focus specifically on female researchers in AI or researchers in STEM – or a combination or AI is assumed as part of STEM?  Clarification of this will highlight more explicitly the unit of analysi

**Literature Review**

 The section titled *Underrepresentation of women in AI* is very effective in establishing a factual context and the identification of the characteristics of the underrepresentation which are consistent across females in paid employment and those in the researcher domain and highlights the continued gap between males and females.  

 The analysis on the challenges presents a good deeper level analysis of the less obvious factors and brings to the fore novel topics for consideration for females in AI.  For instance, the discussion on issues and challenges relating to the lack of attainable role models is effective as it reduces the more general focus on having role models which may not be attainable – and thus have less impact.

 Additionally, factors such as ‘survivorship bias’ – which is less mentioned in research is an important and value-added point in this research. Further the discussion and points made about the focus should not be solely on how to bring more people into STEM but also to develop them and broadening it beyond the female population provides a useful point for debate and consideration.

 Some points to consider are as follows;

- Data and Statistics provided – are these specific to female researchers or females in general in research and non-research fields – while the general data is useful as a framing context if not specific or reflective AI researchers then commentary to this effect is important and assessment of the implications or learnings from the data for female researchers in AI.
- Figure 2. Women bestowed European Research Council Grants in Physical Sciences and Engineering and elevated to IEEE Fellow - is this in general across STEM or AI?
- Linkage of STEM initiatives and those in AI – are the two sets of intiaitves similar and can the synergies of both be aligned to reduce potential duplication or overlap.
- What is the rationale for introducing Marie Curie funding as opposed to similar research funding providers – why this source and is it more relevant or targeted to AI?  Does it provide a fair or appropriate representation of women in broader arena of AI?

More general points relate to metrics applied Metrics to measure and access impact – is it publications; working in AI; researching in AI and or commercialising research and why those?

**Methodology**

 The methodology is not referred to in the article and this should be addressed and take into consideration the following points.

- What is the research approach or methodology adopted – is it the case study method where the cases are the workshops?. Details on the research approach adopted and the rationale for its choice requires consideration.
- Was the workshop designed to investigate or explore the research aims of this paper or was it already available and used for this paper?
- What was the aim and outcomes or learnings that guided the development of the workshop and how do these relate to providing insights into the research questions.  These are important to establish the foundation on which to measure the impact or success of the workshop in delivering on the objectives.
- In relation to measuring or assessing the impact of the workshop – how was the outcomes measured from the different perspectives- what criteria were applied and who undertook the assessment?
- The workshop is presented as leading-edge investigations of AI techniques applied to the fight against COVID-19 pandemic – so was the dominant focus on the description of the projects and their use and benefits in Covid or did the speakers reflect and story tell their personal STEM journey in research and about bringing the research to reality or a finished product/service?
- Reference is made that the AI4FA COVID-19 workshop ‘ *helps in celebrating leading women researchers in AI, and in put a face on *them’ *– *was there a discussion on how the content addressed the personal challenges or the journey the female speakers navigated to *– *did they discuss or refer to the issues identified in the previous sections in  the literature and did they speak to these to enable other females understand and identify ways of overcoming these challenges.
- The discussion on the workshop is largely descriptive and lacks constructive critical analysis or critique to highlight the learnings both positive and negative and what they would do the next time around to add to it – the transferability of the learnings to enable readers to be in a position to develop similar workshops.
- Was the evaluation of the workshop based on the organisers own views or did it engage with participants and speakers to ascertain their feedback – this would provide more objective ‘other and user’ party evaluation to determine where and how the workshop provided ways or mechanisms to address the challenges identified in the review of the literature?
- What criteria or measures were used to determine the impact of the workshop, what were the reasons it was deemed impactful – more constructive critique of the event based on its objectives and in particular to the research questions of this piece.
- What was the rationale for the selection of these speakers and were they considered attainable role models? ‘

**Conclusion**

 In the discussion in the conclusion section reference is made to ‘number of females publishing’ but is this a fair or accurate measure/indicator of ‘gender imbalance in this field’ as the researchers may be productive in achieving outcomes in different areas?

 Content in this section should more explicitly highlight the various contributions the research has made to practice, scholarly and if possible policy or educational domains given the nature of the research questions. The research should speak to those parties/stakeholders who have potential to remedy the challenges identified.

 State future directions for research as these are not mentioned.

Is the topic of the opinion article discussed accurately in the context of the current literature?

Yes

Are arguments sufficiently supported by evidence from the published literature?

Yes

Are all factual statements correct and adequately supported by citations?

Partly

Are the conclusions drawn balanced and justified on the basis of the presented arguments?

Partly

Reviewer Expertise:

Entrepreneurship, EntreSTEAM, Women in STEM, Commercialisation of Research

I confirm that I have read this submission and believe that I have an appropriate level of expertise to confirm that it is of an acceptable scientific standard, however I have significant reservations, as outlined above.

# Reviewer report: Reviewer response for version 1

**DOI:** 10.5256/f1000research.135724.r171783

**Published:** 2023-11-30

**Authors:** Elena De Gioannis

**Affiliations:** 1 University of Milan, Milan, Italy

This article aims to stress the importance of female representation in STEM, focusing in particular on the "new" sector of artificial intelligence. 

 The issue discussed in the article is relevant, as current research on the underrepresentation of women in STEM usually focuses on the engineering sector. However, I think that the authors did not discuss the issue in a proper way.

 In my opinion, there are several limitations that make the article not suitable for publication. First, the underrepresentation of women in AI, which should be the focus of the article, is very briefly presented, while more space is given to the gender gap in other scientific sectors. 

 Second, I think that the description of the four scientists mentioned in the paper does not contribute to the paper. Here, the discussion on the AI4FA COVID-19 conference is said to be an example of a more balanced participation between genders. However, I do not see how a lengthy description of this conference and its speakers can contribute to the understanding of the theme. I do understand the intent of the authors of providing positive examples, however this should have been included in a more rigorous discussion on the gender gap in AI, which in my opinion is missing. 

 A solid background and explanation of the motivations for the description of the conference is missing and, consequently, the conclusions drawn are not justified.

Is the topic of the opinion article discussed accurately in the context of the current literature?

No

Are arguments sufficiently supported by evidence from the published literature?

No

Are all factual statements correct and adequately supported by citations?

Partly

Are the conclusions drawn balanced and justified on the basis of the presented arguments?

No

Reviewer Expertise:

STEM gender gap

I confirm that I have read this submission and believe that I have an appropriate level of expertise to state that I do not consider it to be of an acceptable scientific standard, for reasons outlined above.

# Reviewer report: Reviewer response for version 1

**DOI:** 10.5256/f1000research.135724.r187756

**Published:** 2023-09-04

**Authors:** Lubna Daraz

**Affiliations:** 1 School of Library and Information Science, University of Montreal, Montreal, Canada

Introduction: This article focuses on the crucial and pressing issue of gender imbalances within the AI ecosystem. The authors' primary objective is to emphasize the significance of enhancing the visibility and recognition of women researchers in the field. Within the article, various challenges that women face, such as the "Marie Curie complex," survivorship bias, and imposter syndrome, are briefly discussed. Additionally, the authors present a list of female researchers in AI in Europe and highlight their contributions to COVID-19 research to bolster their arguments.

 Review and Comments:

1. Relevance and Scope: The article tackles a topic of great relevance within the STEM community. Nevertheless, there is a concern about potential bias within the article itself. While the authors commendably cite four notable female AI researchers involved in COVID-19 research, the omission of examples from North America and other regions is notable. It would strengthen the article's credibility to provide a broader international perspective on female role models in AI. I would like reference to a relevant article addressing gender parity in AI within the Canadian context, which is unmentioned in this piece (link provided: https://pubmed.ncbi.nlm.nih.gov/35865270/) ^1^. (Conflict of Interest is declared by the reviewer). Therefore, it is recommended that the article be revised to explicitly state its regional context, specifically focusing on Europe.
2. Promotional Aspect: There appears to be a promotional undertone throughout the article, primarily for the Workshop AI4FA COVID-19 and its keynote speakers. To maintain a balanced and objective tone, it is advisable to minimize the detailed information about the workshop and the speakers. A brief mention and link to the workshop can suffice, ensuring that the primary focus remains on the broader issues of gender imbalances in AI.
3. Structural and Professionalism Improvements: Several structural and professionalism issues require attention. First, there are broken links within the article, which hinder the reader's experience and access to relevant resources. These should be promptly fixed to ensure a seamless reading experience. Furthermore, both the abstract and the article's content could benefit from a more professional and standard article format, enhancing the overall presentation.
4. Avoiding Bias: The central idea presented in the article, while important, carries the risk of inherent bias. It is crucial to present a balanced perspective and acknowledge the progress and initiatives addressing gender imbalances in AI alongside the challenges. Striving for objectivity and comprehensiveness will enhance the article's impact and credibility.

Conclusion:

 In conclusion, the article addresses a significant and timely issue regarding gender imbalances in the AI ecosystem. However, it requires substantial revisions to ensure impartiality, broaden its geographical scope, improve its structure and professionalism, and mitigate potential biases. By taking these recommendations into account, the authors can create a more impactful and inclusive article that contributes effectively to the ongoing conversation on gender equity in AI.

Is the topic of the opinion article discussed accurately in the context of the current literature?

Partly

Are arguments sufficiently supported by evidence from the published literature?

Partly

Are all factual statements correct and adequately supported by citations?

Yes

Are the conclusions drawn balanced and justified on the basis of the presented arguments?

Partly

Reviewer Expertise:

Implementation Science, Health Informatics, Health Literacy, Internet, Diverse populations, Social determinants of health, Health equity.

I confirm that I have read this submission and believe that I have an appropriate level of expertise to confirm that it is of an acceptable scientific standard, however I have significant reservations, as outlined above.

## References

[1] : Inferior: The Challenges of Gender Parity in the Artificial Intelligence Ecosystem-A Case for Canada. *Front Artif Intell* .2022;5: 10.3389/frai.2022.931182 931182 10.3389/frai.2022.931182 35865270 PMC9295739
